# The Management and Prevention of Delirium in Elderly Patients Hospitalised in Intensive Care Units: A Systematic Review

**DOI:** 10.3390/nursrep14040219

**Published:** 2024-10-15

**Authors:** Sarai Zaher-Sánchez, Pedro José Satústegui-Dordá, Enrique Ramón-Arbués, Jose Angel Santos-Sánchez, Juan José Aguilón-Leiva, Sofía Pérez-Calahorra, Raúl Juárez-Vela, Teresa Sufrate-Sorzano, Beatriz Angulo-Nalda, María Elena Garrote-Cámara, Iván Santolalla-Arnedo, Emmanuel Echániz-Serrano

**Affiliations:** 1 Miguel Servet Hospital, Aragonese Health Service, 50017 Zaragoza, Spain; szaher@salud.aragon.es; 2 SAPIENF Research Group, Department of Physiatry and Nursing, Faculty of Health Sciences, University of Zaragoza, 50009 Zaragoza, Spain; pjsd@unizar.es (P.J.S.-D.); eramon@usj.es (E.R.-A.); jaguilon@unizar.es (J.J.A.-L.); spperezc@unizar.es (S.P.-C.); eechaniz@unizar.es (E.E.-S.); 3 Faculty of Health Sciences, San Jorge University, Villanueva de Gállego, 50830 Zaragoza, Spain; 4 Faculty of Medicine, University of Salamanca, 37007 Salamanca, Spain; jasalao@usal.es; 5 Health and Healthcare Research Group (GRUPAC), Department of Nursing, Faculty of Health Sciences, University of La Rioja, 26006 Logroño, Spain; maria-elena.garrote@unirioja.es (M.E.G.-C.); ivan.santolalla@unirioja.es (I.S.-A.); 6 San Pedro Hospital, Rioja Health Service (SERIS), 26001 Logroño, Spain; bangulo@riojasalud.es

**Keywords:** delirium, acute confusion, intensive care unit, ICU, prevention, treatment, drug therapy, nursing, interventions, management, aged, elderly

## Abstract

Background: Delirium or an acute confusional state (ACS) is characterised as being a frequent and complex hospital complication in older adult patients, which can affect their level of independence and increase patient morbidity and mortality. Critically ill patients in the intensive care unit (ICU) frequently develop ICU delirium, leading to longer hospital and ICU stays, increased mortality and long-term impairment. Objectives: This review aims to assess existing evidence of interventions that can be considered effective for the management and prevention of delirium in ICUs, reducing short-term morbidity and mortality, ICU and hospital admission times and the occurrence of other long-term complications. Methodology: For this systematic review, we searched Medline, PubMed, Cochrane Library, CINHAL, LILACS, SciELO and Dialnet from January 2018 to August 2024, in English, Spanish and French. MeSH descriptors were adjusted to search the different databases. We also checked Prospero for ongoing systematic reviews. Main results: The electronic search yielded a total of 2656 studies, of which 14 trials met the eligibility criteria, with a total of 14,711 participants. We included eight randomised clinical trial (RCTs), four cohort analyses, one systematic review and one observational trial, including participants over 65 years admitted to the ICU. Ten of these studies were based on pharmacological interventions, three of them examined non-pharmacological interventions and the remaining study examined mixed (pharmacological and non-pharmacological) interventions. Six placebo RCTs were included, plus four reported comparisons between different drugs. Regarding non-pharmacological interventions, nursing programmes focused on optimising modifiable risk factors or the use of therapies such as bright light are emerging. Regarding mixed interventions, we found the combination of invasive techniques and with sedoanalgesia. Conclusions: Due to its satisfactory level of sedation, dexmedetomidine is presented as a viable option because, although olanzapine offers safer results, postoperative administration angiotensin inhibitor systems significantly reduced the incidence of delirium. As for propofol, no significant differences were found. Among the non-pharmacological and mixed therapies, bright light therapy was able to reduce the incidence of delirium, and the combination of epidural/general anaesthesia was effective in all subtypes of delirium. Concerning the remaining interventions, the scientific evidence is still insufficient to provide a definitive recommendation.

## 1. Introduction

Delirium or acute confusional syndrome (ACS) is defined, according to the Di-agnostic and Statistical Manual of Mental Disorders (DSM-V) [1], as an impairment of attention, awareness and cognition, capable of reducing the ability to direct, focus, maintain and shift attention and reduces orientation in the environment. This disturbance develops over a short period of time, fluctuates and represents an acute change in attention and awareness from the individual’s baseline state [1,2]. Three types of psychomotor delirium have been described: hypoactive, hyperactive and mixed delirium [3].

ACS should be considered a medical emergency and early diagnosis of ACS makes it possible to prevent its consequences and complications. Diagnosis is based on a review of the clinical history and an adequate and complete anamnesis, and the use of complementary tests and scales. In recent years, numerous diagnostic tools have been designed to address the under-diagnosis of delirium. One of the most widely used instruments for the diagnosis of delirium is the Confusion Assessment Method (CAM) [4,5], which was later adjusted and validated as a version designed for Critical Care Units (CCUs), known as the Confusion Assessment Method for Intensive Care Units (CAM-ICU) [6].

ACS is generally a multifactorial syndrome in which predisposing factors (hospitalisation, comorbidity, polypharmacy, sensory deprivation, age > 65 years, etc.) and precipitating factors (drug use or deprivation, iatrogenic processes, environmental factors, etc.) are present [7].

ACS is a phenomenon that is increasingly observed in hospitalised older adults. With respect to hospital admissions, approximately 10–30% of patients will develop ACS during hospitalisation [8]. The incidence and prevalence of delirium vary according to age, type of patient and place of hospitalisation [7]. In hospitalised persons over 65 years of age, an incidence of 10–40% is observed [9]. In postoperative patients, the incidence of ACS is close to 10%, while in more complex procedures such as heart surgery with extracorporeal circulation, or in orthopaedic procedures such as hip surgery, it can exceed 50%. In older adults admitted from the emergency department, the incidence of ACS is 10%, while in the same population group admitted to the critical care unit requiring mechanical ventilation it is 50–80% [10,11].

Researching age, Flórez and Velázquez found a statistically significant trend between the development of ACS and older people, i.e., the risk of developing delirium is five times higher in patients aged ≥85 years than in patients aged 65–74 years.

The onset of ACS during the hospitalisation period has a serious impact on patients, as this syndrome is associated with a significant risk of cognitive, functional and quality of life impairment in the short and long term. Furthermore, the development of delirium is associated with longer hospitalisation times, longer average ICU stays, high healthcare costs and morbidity and mortality. There are studies linking delirium with higher in-hospital mortality (26.7% vs. 21.4%) and higher ICU mortality (19.7% vs. 10.3%) [12]. With regard to mortality risk, studies show that the risk trebles in the 6 months following the development of ACS [13]. In terms of the time required for admission, there are similar studies that report that the average ICU stay is 9 days compared to 4 days for patients who do not develop ACS [14], while hospital stays amount to 21 days compared to the initial 11 days [6]. Healthcare costs can be up to 59% higher in cases where delirium occurs during the time of admission and can double in the year following hospitalisation [15,16].

This review aims to determine which interventions could be considered effective in the management and prevention of delirium in ICUs, reducing short-term morbidity and mortality, ICU and hospital admission times and the occurrence of other long-term complications. The identification of these interventions will facilitate the development of interdisciplinary protocols to standardise day-to-day clinical practise in the management of delirium.

## 2. Methods

We carried out a systematic review to determine which pharmacological and non-pharmacological interventions could be considered effective in the prevention and management of ACS in older adults admitted to intensive care units, and to evaluate the existing evidence on the effect of these interventions on the development of the syndrome and their impact on reducing mortality and decreasing the time spent in ICUs and other complications associated with the syndrome.

This work was registered in the International Prospective Register of Systematic Reviews (PROSPERO). Registration code CRD42024501365.

### 2.1. Formulation of the Research Question

The research question was defined by following the PICO (Patient, Intervention, Comparison and Outcome) structure for clinical questions model [17] (Table 1).

### 2.2. Criteria for Inclusion/Exclusion of Studies in This Review

The search strategy designed was carried out between July and August 2024, with the aim of locating all studies that met the inclusion criteria. Randomised clinical trials (RCTs), systematic reviews, cohort studies and case–control studies published between January 2018 and August 2024, written in English, French and Spanish, were included. Regarding the target population, studies of patients admitted to ICUs aged 65 years or older were included. Studies of both medical and surgical intensive care services were included. Studies with intubated and non-intubated patients were included. Regarding the type of interventions, we included studies with pharmacological or non-pharmacological interventions or the administration of both to prevent or manage delirium in ICUs. Regarding the types of outcome measurements used to assess the efficacy of the interventions, we used primary outcomes, an assessment of the rates of the development of delirium in ICUs, the duration of delirium once onset, the reduction in short-term morbidity and mortality secondary to the development of delirium; and secondary outcomes, reduction in time spent in critical care units and hospital admissions and the development of other long-term complications or adverse events associated with the development of this syndrome. As exclusion criteria, non-randomised clinical trials, cross-sectional studies and expert opinions were excluded and studies with conflicts of interest and questionable methodological quality were excluded. With regard to the target population, patients with any other form of mental disorder, such as dementia, were excluded.

### 2.3. Search Strategy

The search included the following electronic bibliographic databases: Medline, Cochrane Library, CINHAL, LILACS, SciELO and Dialnet. The research was also carried out using the database aggregator PubMed. Prospero was also consulted for an overview and comparison of completed or ongoing reviews and meta-analyses related to our study topic. Subsequently, we carried out another manual search using external sources such as Google Scholar and other bibliographic resources available in the library of the University of Zaragoza (AlcorZe). We used the following MeSH and DeCS keywords and descriptors for the search: delirium, acute confusion, intensive care unit, ICU, prevention, treatment, drug therapy, nursing, interventions, management, aged and elderly, which were linked to various Boolean operators. We then reviewed the references of the selected articles and searched for bibliographic citations to identify additional studies. Table 2 shows the search strategy.

The article selection process was carried out using Covidence software. Covidence is a screening and extraction tool that, among other functions, enables users to upload search results, filter abstracts and full-text study reports, complete data collection, perform risk of bias assessments and resolve disagreements. The initial search was conducted by one researcher. In order to eliminate duplicates and the review of titles and abstracts, two independent peer reviews were used to confirm the inclusion and exclusion criteria. Articles selected by at least one of the reviewers were analysed in full text for re-evaluation. If there were discrepancies, a third reviewer was asked to act as arbitrator. The inclusion of results was carried out independently by two reviewers. The existing references in the included papers were then analysed. The analysis of the methodological quality and the extraction of data from the articles were carried out independently by two people. Discrepancies resulting from the work of the two reviewers were independently refereed by a third reviewer.

## 3. Results

**Figure 1:**
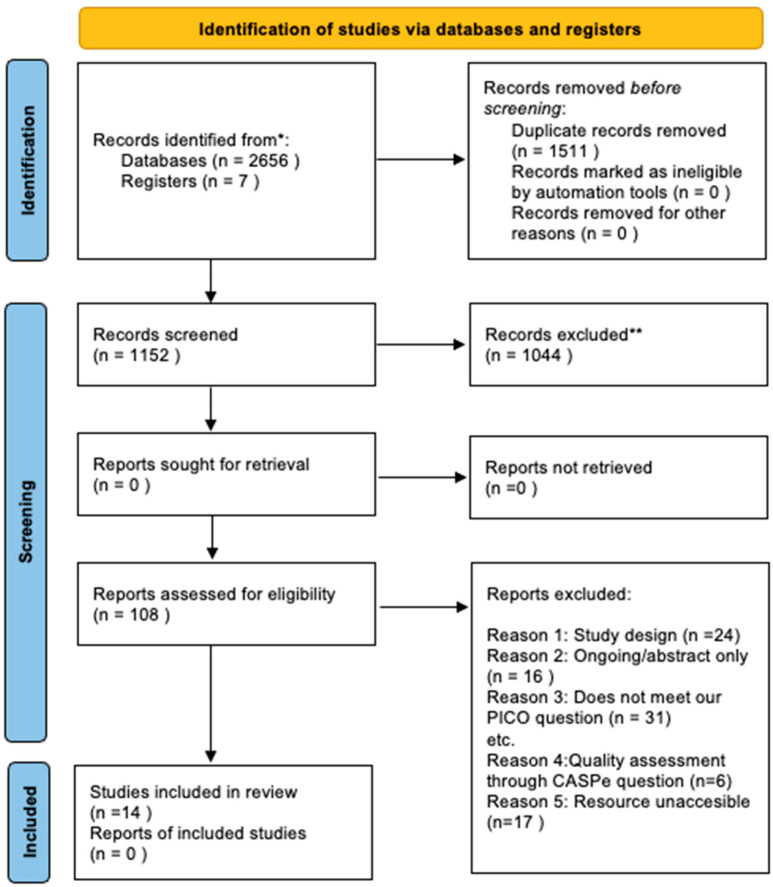
PRISMA study selection flowchart. * Consider, if feasible to do so, reporting the number of records identified from each database or register searched (rather than the total number across all databases/registers). ** If automation tools were used, indicate how many records were excluded by a human and how many were excluded by automation tools.

The researchers retrieved a total of 2656 articles (PubMed 1623, Medline 629, Cochrane 15, CINAHL 96, LILACS 27, Dialnet 11, SciELO 1, Prospero 257). After removing duplicate articles and including 7 articles from additional sources, 1152 results were analysed by title and abstract, from which 108 articles were selected. The inclusion and exclusion criteria described above were then applied, and 14 papers emerged as relevant, which were then critically analysed. The procedure followed in this “systematic review” is described in the PRISMA flowchart [18] (Figure 1). At each stage, the three reviewers applied measures to minimise the risk of bias and error.

### 3.1. Quality Assessment

To ensure methodological quality, the instrument used to assess the quality of the finally chosen studies was the CASPe critical reading tool [19]. Two reviewers conducted an independent assessment of the quality of the articles using the CASPe (Critical Appraisal Skills Programme Español) critical reading tool [19]. Each of the assessment tools included between 10 and 11 questions that assigned an overall quality rating to each study. These ratings ranged from low quality (score below 40%) to medium quality (up to 70%) and high quality (over 70%). In the case of a disagreement, a consensus was reached through discussion with a third reviewer. At this stage of the research, the team agreed to exclude four papers with a quality level below 40%. The overwhelming majority of the included reviews (n = 8) were rated as “high” quality, and the remaining papers (n = 4) were rated as “medium” quality. The most common caveats were related to not detailing the review question and a lack of methods to minimise potential risks of bias and error during data collection.

In the end, we included 14 studies in this systematic review, with a total of 14,349 participants [20,21,22,23,24,25,26,27,28,29,30,31]. In terms of the type of study included, four of the studies were cohort studies, seven were randomised critical trials and one was a systematic review.

### 3.2. Place and Period of Study

The 14 studies were conducted between 2011 and 2024; and originated from the USA [21,27,30,32], the Netherlands [20,28,31], China [22,23], Belgium [24], Egypt [29], France [33] and Thailand [26]. All the studies found declared no conflicts of interest.

The following table (Table 3) includes the data obtained from the studies selected for this review and the information gathered is divided into the following elements: author, country and year of publication; type of study, sample size, interventions included, variables measured, scales used, main results obtained and the CASPe score obtained after evaluating each study included:

### 3.3. Interventions

The pharmacological interventions included haloperidol, olanzapine, valproic acid, acetaminophen, melatonin, angiotensin converting enzyme inhibitors and angiotensin receptor inhibitors. Sedation interventions using dexmedetomidine or propofol appeared, the infusion of which was stopped in the morning for reassessment of the patient’s condition. Mixed anaesthesia induction procedures such as general anaesthesia or a combination of general anaesthesia and epidural anaesthesia appeared. Other studies included non-pharmacological interventions, related to environmental therapies such as bright light therapy or music intervention or a preventive nursing care plan.

Some pharmacological interventions were compared with placebo or standard ICU care, and the latter were not described in detail.

### 3.4. Types of Participants

Five of the studies included in this review included patients aged ≥18 years [20,21,27,28,31]. Another article began by including individuals ≥50 years in its sample [26], while in five other studies the age of the sample was ≥60 years [22,24,29,30,32]; nevertheless, in all of the studies mentioned above, the final mean age of the sample obtained exceeded the age of our target population (≥65 years). Only one of the studies studied a specific elderly population, ≥75 years [23].

All of the selected articles suggest the existence of some pre-existing cognitive impairment as an exclusion criterion in their studies.

In terms of patient type, there are patients who developed specific pathologies or who underwent specific surgeries such as cardiopulmonary bypasses [24,30] or cardiac interventions [29,33].

### 3.5. Summary of the Results Obtained

To summarise the results obtained in the following review, the findings will be presented according to the type of intervention, leading to pharmacological, non-pharmacological and mixed interventions.

### 3.6. Pharmacological Interventions

In general, pharmacological interventions did not provide consistent results in terms of delirium prevention and symptomatology control, nor did they improve mortality outcomes, length of hospital and ICU admission or maintenance of cognitive function.

Most of the selected articles were generally based on people without delirium or a high risk of developing delirium, offering preventive pharmacological alternatives; only one of the studies compared the effect of dexmedetomidine to olanzapine in the control of delirium in patients who met DSM-V diagnostic criteria for ACS [23].

Among the pharmacological alternatives, there is a predominance of studies aimed at analysing the efficacy of dexmedetomidine, whether in a single treatment, in combination with other drugs or by comparing efficacy between drugs. Liu et al. [23] suggest making a comparison between intravenous dexmedetomidine and oral treatment with olanzapine in patients without surgical intervention or mechanical ventilation in ICUs; in this article dexmedetomidine is shown to be a viable option as it achieved a more satisfactory sedative effect and showed greater effectiveness in the control of delirium than olanzapine. In contrast, olanzapine offered safer results as dexmedetomidine was associated with a higher number of adverse events such as increased intubation rates and length of hospital admission, and the study concluded that, in patients without mechanical ventilation, olanzapine administration was safer in controlling delirium than dexmedetomidine.

In line with this, Momeni et al. [24] and Pereira et al. [25] suggest comparing sedative interventions using propofol and dexmedetomidine. While Momeni [24] focused only on patients undergoing cardiac surgery with cardiopulmonary bypass, Pereira [25] included any patient admitted to the medical or surgical ICU. However, both had one main focus in common: the incidence of delirium. Momeni [24], comparing the administration of propofol and dexmedetomidine perfusion vs. placebo, reported that although the propofol/dexmedetomidine combination was associated with a decrease in the mean length of admission compared to the placebo, there was no significant difference in the incidence of delirium between the two interventions. Pereira [25], in contrast, compared the administration of propofol with dexmedetomidine, in order to discover which drug reduces the incidence of delirium. This study suggests that dexmedetomidine sedation is associated with a lower incidence of delirium in ICUs compared to the placebo, as well as with a lower risk of developing side effects such as bradycardia or hypotension, although this needs to be studied further in order to provide a more confident recommendation. Huet [33] concluded that nocturnal administration of dexmedetomidine failed to decrease the incidence of postoperative delirium after cardiac surgery. All of the above infusions were temporarily interrupted for the re-evaluation of the patient, discontinued in the event of the end of delirium or restarted in the event of ongoing or worsening of delirium.

In contrast, Subramaniam et al. [30], limited by a significantly small sample size (n = 121 participants), compared the effect of acetaminophen vs. a placebo combined with propofol and dexmedetomidine and concluded that the incidence of delirium, duration of delirium, length of admission and use of other analgesics was significantly lower in the group receiving acetaminophen. There was no statistically significant difference between the use of dexmedetomidine and propofol.

Duprey et al. [20] and Van Den Boogard et al. [31], who tested haloperidol against a placebo in the prevention of ICU delirium, found no effect on in-hospital mortality, number of delirium- and coma-free days, length of ICU stay or number of ventilator-free days. Duprey’s study [20] concluded that the effect of haloperidol was dose-dependent and decreased over time, as in patients with delirium or a high risk of delirium an association was observed between mortality and haloperidol administration, with the risk of mortality during the first 28 days being lower than during the first 90 days. In patients without delirium, haloperidol did not prevent the development of delirium or reduce the risk of mortality. However, Van Den Boogard [31] found no significant difference in survival during the first 28 and 90 days in patients who received prophylactic doses of haloperidol compared to those who received a placebo.

There were other pharmacological lines of research, including the use of melatonin, valproic acid and angiotensin-converting enzyme inhibitors and angiotensin receptor blockers. Farag et al. [21] concluded that preoperative use of angiotensin inhibitor systems made no significant difference in the incidence of delirium, but in contrast, postoperative use halved the incidence of delirium. In the study by Quinn et al. [27], it was concluded that, although more studies are needed to provide a more confident recommendation, the use of valproic acid could be seen as a viable option in the resolution of delirium and in the tapering of other antipsychotic medications, as well as being associated with minimal side effects.

### 3.7. Non-Pharmacological Interventions

The UNDERPIN-ICU programme developed by Rood et al. [28], which focused on reducing all modifiable risk factors such as visual and auditory impairment, disorientation, sleep deprivation, mobility and cognitive impairment and which had already been shown to be effective in non-ICU patients, showed no change in the number of days without delirium or coma.

In contrast, Potharajaroen et al. [26] studied the efficacy of using bright light therapy (5000 lux for 2 h) versus usual care (undefined) and exposure to a 500 lux light source. This therapy significantly reduced the incidence of delirium by improving circadian rhythms and sleep/wake cycles.

Johnson [32] proposed the use of pre-selected music to prevent delirium, addressing the pathopshysiologic mechanisms that contributes to delirium versus the usual care. Music contributes to the reduction in acute physiologic stressors, reducing, therefore, the incidence of delirium.

### 3.8. Mixed Interventions

Following this line of combining pharmacological and non-pharmacological interventions, a combination of epidural/general anaesthesia has been compared to general anaesthesia in patients undergoing major non-cardiac thoracic or abdominal surgery [22].

Li et al. [22] carried out an assessment of the patient’s baseline condition prior to surgery to evaluate the impact of the development of delirium on the patient’s ability to perform basic activities of daily living and cognitive function. The incidence of delirium in the first 7 days post-surgery was significantly lower in patients assigned to the epidural/general anaesthesia combination. This reduction was common to all pre-defined subtypes of delirium.

## 4. Discussion

The main objective of this review was to analyse the available literature on the effect of pharmacological and non-pharmacological interventions, or the combination of both, in the prevention and management of ACS in patients aged ≥65 years admitted to intensive care units, in medical or surgical patients.

We conducted a broad search which yielded numerous potentially eligible studies, suggesting that there is considerable interest in identifying and studying effective strategies and therapies for the treatment of delirium. Fourteen studies were included in this systematic review, answering our PICO questions and fulfilling our inclusion and exclusion criteria and covering a total of 14,711 randomised ICUs patients.

The selected studies carried out different interventions including pharmacological therapies, sedation administration, as well as non-pharmacological therapies aimed at reducing modifiable risk factors for delirium, such as environmental interventions, physical therapies or preventive nursing care. These included interventions compared to a placebo, standard ICU care and standard sedation processes.

The quality of these studies was moderate and was assessed using the CASPe methodological quality tool [19].

As for pharmacological interventions, they indicate no consistent results for preventing delirium or improving outcomes such as length of hospital and ICU stay, incidence of delirium, reducing the delirium rate or limiting long-term complications.

In line with our research, Skrobi et al. [34] studied the effects of using low-dose nocturnal dexmedetomidine in critically ill patients. Although this study does not meet our methodological requirements for the type of patient, the results show that the use of dexmedetomidine significantly reduces the risk of delirium among ICU patients as well as reducing the length of stay.

In recent years, various bundles of care approaches to prevent and manage delirium have been published. For example, including early mobilisation, family engagement, deprescribing to avoid oversedation or reducing physical restraint [35,36,37].

Regarding the limitations of the chosen studies, there is much debate about the assessment of delirium in the ICU and the diversity of scales and instruments used. Although all used the Confusion Assessment Method for the Intensive Care Unit (CAM-ICU), only four studies exclusively used this scale [21,27,28,29,32,33]. Other rating scales were used such as Richmond Agitation Sedation Scale (RASS) [20,22,23,24], Confusion Assessment Method (CAM) [25,30,31] and the Insomnia Severity Index (ISI) [26].

The use of different definitions of delirium naturally limits the robustness of the conclusions that can be drawn from this review. It is also important to agree on primary and secondary outcomes, as delirium should be assessed as a primary outcome in future studies. A consensus between the tools used in the ICU for delirium screening and the definition of delirium would allow more rigorous results to be obtained.

Another limitation we observed was the absence of a study of delirium focusing on the previously defined subtypes (hypoactive, hyperactive or mixed). Only one mention of these subtypes was found in the article by Li et al. [22], in which a similar effect of combined epidural/general anaesthesia on all subtypes of delirium is suggested.

Another limitation we found in our review is the presence of different sedation and drug dosing protocols, as well as significant differences in the type of patients and patient characteristics. Due to high heterogeneity, results should be interpreted with caution.

We examined completed or ongoing systematic reviews in PROSPERO that studied interventions similar to those included in this study and found comparisons between pharmacological interventions using dexmedetomidine or propofol as sedative options in the management of delirium in critically ill patients over 65 years of age; we also found reviews comparing only non-pharmacological interventions in the prevention and management of delirium. However, no recent review was found that included both interventions together with the combination of both.

We acknowledge that there is an increasing interest in the topic and future studies are underway as we have identified sixteen ongoing studies, of which, nine have a large target enrolment number, suggesting a growing interest in treating ICU delirium among the elderly. For example, as we searched the evidence available for this review, we spotted several ongoing studies through clinical trials, including numerous pharmacological and non-pharmacological interventions like the ones included in this review. These studies should strengthen our results once they are published, and they may alter the direction of our findings.

### Implications for Research

The clinical benefits of dexmedetomidine, as well as different approaches to sedation intervention such as overnight infusion and the daily interruption of sedation, mixed interventions and early physical/cognitive approach or the employ of environmental interventions for the management and prevention of delirium are still unclear and require further investigation.

The potential of non-pharmacological therapies such as environmental interventions should also be further explored.

Future studies should be designed to include similar outcomes such as short/long-term function, quality of life and the effects of delirium on morbidity and mortality. Ideally, studies should include large samples to facilitate an analysis of the effects of treatments on the subtypes of delirium.

There is also a need for consensus regarding the assessment of delirium in the ICU and the diversity of scales and instruments used, as well as the predefined outcomes. A difference in sleep-related outcomes may have been observed as some of the studies included did not evaluate sleep quality as an outcome in preventing delirium.

Short- and long-term cognitive function, physical function and the quality of life of patients need to be studied and compared with the patients’ baseline functions before admission to ICUs and the development of delirium. There is also a need to standardise drug patterns in order to correctly identify the benefit of the drug in question.

## 5. Conclusions

Delirium is one of the most frequent manifestations of cognitive dysfunction in critically ill people, and its diagnosis is often underestimated, leading to a worse prognosis and having negative repercussions on economic and health matters.

From this review, we can conclude that optimal sedation appears to have been achieved more readily with dexmedetomidine. With regard to the use of dexmedetomidine, we found evidence that the use of this drug would be feasible after achieving satisfactory sedation levels and has a greater effectiveness in the control of delirium than olanzapine. However, it has also been considered to be a drug with a lower safety index because it has been associated with higher risks adverse events such as hypotension; although it had to be temporarily held in some instances because of bradycardia and/or hypotension, no patients were removed from the studies due to safety issues. No significant differences in the incidence of delirium were found between the use of propofol and dexmedetomidine.

Based on a single study and a very small sample, the benefits of acetaminophen on the incidence and duration of delirium appear inconclusive. The effect of valproic acid needs further study before a confident recommendation can be made. The use of angiotensin-inhibiting systems was only effective if they were administered during the postoperative process. The current limited evidence indicates that the use of haloperidol, despite being one of the most commonly used practises in the critical care unit, cannot be considered an effective strategy

The clinical benefits of haloperidol, acetaminophen or other physical, cognitive or environmental interventions for the prevention and management of delirium are unclear and warrant further investigation in large multicentre studies.

The UNDERPIN-ICU nursing intervention did not prevent delirium, nor did it improve any of the proposed secondary outcomes. However, bright light therapy did significantly reduce incidence due to an improvement in sleep–wake cycles, as was the case for the usage of music that soothes patients in stressful situations and, therefore, reduces the incidence of delirium.

The epidural/general anaesthesia combination was effective in all subtypes of delirium and significantly reduced the incidence of delirium during the first 7 days.

Despite the above, few of these measures are used in intensive care units and the implementation of these strategies for the prevention, monitoring and management of delirium should be a priority for nursing research in this environment. The scientific evidence on prevention and treatment is still insufficient to reach a definitive conclusion, and therefore new lines of research are required in order to improve knowledge and skills for the care of people suffering from this type of disorder.

## Figures and Tables

**Table 1:** Formulation of the PICO question.

P (Patient, Population)	I (Intervention)	C (Comparison)	O (Results, Outcomes)
Persons admitted to critical care units aged 65 or older	Pharmacological and non-pharmacological measures in the prevention and management of delirium	Standard interventions in non-specific ICU care for the treatment of delirium.	Identifying those interventions capable of reducing the complications associated with the development of delirium, such as the reduction in entry time or the reduction in morbimortality.

**Table 2:** Articles obtained by database, descriptors and Boolean operators used.

Source	Search Strategy	Filters	Results	Selected Articles
	((delirium OR acute confusion)			
	AND icu AND prevention) [MeSH Terms]		197	1
	((delirium OR acute confusion)			
	AND icu AND treatment) [MeSH Terms])		536	2
PubMed	(delirium OR acute confusion) AND icu AND (treatment OR management) [MeSH Terms]	January 2018–August 2024 Aged (65+ years)	575	3
	(delirium OR acute confusion)			
	AND nursing interventions AND		190	2
	(intensive care unit OR icu) [MeSH			
	Terms]			
	(delirium OR acute confusion)			
	AND drug therapy AND (intensive care unit OR icu) [MeSH Terms]		125	3
Medline	(delirium OR acute confusion) AND icu AND prevention AND (aged OR elderly) (delirium OR acute confusion) AND icu AND (treatment OR management) AND drug therapy AND (aged OR elderly) (delirium OR acute confusion) AND nursing interventions AND (intensive care unit OR icu) AND (aged OR elderly)	January 2018–August 2024	246 136 247	2 1 0
	(delirium OR acute confusion) AND icu AND prevention AND (aged OR elderly)		3	0
Cochrane Library	(delirium OR acute confusion) AND icu AND (treatment OR management) AND drug therapy AND (aged OR elderly)	January 2018–August 2024	6	0
	delirium AND nursing interventions AND (intensive care unit OR icu) AND (aged OR elderly)		6	0

**Table 3:** Evaluation table of the results.

Author, Country and Year	Type of Study	Sample	Interventions	Variables Measured	Scales Used	Main Findings	CASPe Score
Duprey MS, et al. [20]. Netherlands, 2021.	Post hoc cohort analysis of a randomised trial	1495	Comparison of the effect of haloperidol 2 mg, haloperidol 1 mg and placebo in the treatment of delirium, symptomatology and associated mortality.	Incidence of delirium, presence or absence of symptoms, improvement of symptoms and associated mortality.	RASS and CAM-ICU	In patients without delirium on admission to the ICU, haloperidol may be associated with increased survival.	7 points
Farag E et al. [21]. USA, 2020.	Retrospective cohort analysis	4864	Incidence of delirium of patients after pre- and post-surgical treatment with angiotensin-converting enzyme inhibitors or angiotensin receptor antagonists versus those who did not receive them.	Incidence of postoperative delirium, duration of hospital and ICU admission, use of postoperative sedoanalgesia.	CAM-ICU	Preoperative use of the drugs listed above is not associated with a decreased incidence of delirium. Postoperative use is associated with a lower likelihood of developing delirium.	8 points
Li YW et al., [22]. China, 2021.	Randomised clinical trial	1802	Comparison of general anaesthesia with postoperative intravenous analgesia vs. combined general/epidural anaesthesia with postoperative epidural analgesia.	Presence or absence of delirium, duration of delirium, length of hospitalisation and mortality within 30 days after surgery.	CAM-ICU and RASS	A lower incidence of delirium was observed in patients belonging to the combined general/epidural anaesthesia group compared to the general anaesthesia group.	9 points
Liu S, et al. [23]. China, 2021.	Retrospective cohort analysis	263	Treatment with IV dexmedetomidine or OV olanzapine after diagnosis of delirium.	To assess efficacy and safety of dexmedetomidine and olanzapine: level of sedation, drug dose and duration, incidence of combination with sedoanalgesia, adverse effects, intubation rates and prognosis.	CAM-ICU and RASS	Olanzapine was found to be a safer drug, reducing the risk of intubation and ICU admission time; however, dexmedetomidine showed a greater sedative effect.	9 points
Momeni M, et al. [24]. Belgium, 2021.	Randomised clinical trial	420	Comparison of propofol + dexmedet omidine vs. propofol + placebo (saline 0.9%).	Incidence of delirium during hospital admission, duration of delirium, length of ICU and hospital admission, total dose of inotropic and vasopressor drugs and dose of sedoanalgesia administered.	CAM-ICU and RASS	We found no evidence that the addition of dexmedetomidine to the postoperative propofol regime reduces the incidence of delirium.	8 points
Pereira JV, et al. [25]. 2020	Systematic Review	1407	Comparison of dexmedetomidine versus propofol sedation in reducing the incidence of delirium and assessment of associated risks and benefits.	Incidence of delirium, length of ICU and hospital admission, duration of mechanical ventilation, incidence of hypotension and bradycardia.	CAM-ICU and CAM	A lower incidence of delirium in the ICU is associated with the infusion of dexmedetomidine versus the use of propofol.	9 points
Potharaja roen S, et al. [26]. Thailand 2018.	Randomised clinical trial	62	Efficacy of bright light therapy versus exposure to 500 lux light source.	Incidence of delirium in postoperative patients admitted to the ICU.	CAM-ICU and ISI	Bright light therapy may reduce the incidence of delirium by improving sleep–wake disturbances.	7 points
Quinn NJ, et al. [27]. USA, 2021.	Retrospective cohort analysis	80	Efficacy of valproic acid administration in the treatment of delirium or agitation.	Days of ICU admission, incidence of intubation, incidence of delirium after the start of administration and duration of treatment.	CAM-ICU	An improvement in the reduction in delirium and other drug use is suggested; further studies are needed.	7 points
Rood PJT, et al. [28]. Netherlands, 2021.	Randomised clinical trial	1749	Effectiveness of the UNDERPIN-ICU intervention programme, aimed at optimising modifiable risk factors within the first 28 days of ICU admission.	No. of days without delirium and coma, incidence of delirium, duration, mortality during the 28 and 90 days after admission, incidence of reintubation, re-admission to ICU, unplanned removal of tubes and catheters, use of physical restraints, duration of hospital admission.	CAM-ICU	No change in the number of days without delirium or coma during the first 28 days after admission could be determined.	9 points
Shokri H, et al. [29] Egypt, 2019	Prospective observational trial	286	Comparing prophylactic dexmedetomidine in continuous infusion vs. clonidine IV	Incidence of delirium, duration of delirium, ICU stay length.	CAM-ICU	In patients undergoing coronary artery bypass grafting, dexmedetomidine sedation reduced post-surgery control and management of delirium and decreased the length of hospital stay compared to clonidine.	8 points
Subramaniam B, et al. [30]. USA, 2019	Randomised clinical trial	121	To assess the efficacy of the acetaminophen + propofol/dexmedetomidine combination versus placebo.	Incidence of delirium during hospitalisation, duration of delirium, cognitive level at discharge, need for analgesia.	CAM-ICU and CAM	There was a reduction in in-hospital delirium following the use of the combination of acetaminophen with propofol or dexmedetomidine.	8 points
Van den Boogaard M, et al. [31]. Netherlands, 2018	Randomised clinical trial	1789	Comparison of effect of haloperidol 2 mg, haloperidol 1 mg and placebo.	Survival in the first 28 and 90 days after ICU admission, incidence of delirium, number of days without delirium or coma, duration of mechanical ventilation and length of admission.	CAM-ICU and CAM	In patients at risk of delirium, haloperidol treatment did not improve survival in the first 28 days.	8 points
Johnson K, et al. [32] USA, 2018	Randomised clinical trial	40	Comparison of the effect of pre-recorded music listening vs usual care twice a day for 60 min over 3 days.	Incidence of delirium during hospitalisation, duration of delirium.	CAM-ICU	Music had an impact over those pathophysiologic mechanisms that cause delirium, preventing the development of delirium.	7 points
Huet O et al., [33] France, 2024	Randomised clinical trial	333	Comparing the effect of using an overnight infusion of dexmedetomidine postoperative vs placebo for preventing postoperative delirium among patients undergoing cardiac surgery	Occurrence of postoperative delirium, length of ICU stay, length of hospital stay and hospital mortality.	CAM-ICU	Usage of overnight infusion of dexmedetomidine had no significant effect on developing postoperative delirium.	9 points

## Data Availability

Under request first author.

## Public Involvement Statement

No public involvement in any aspect of this research.

## Guidelines and Standards Statement

This manuscript was drafted against the PRISMA for systematic reviews research.
